# Innovation in hyperlink and social media networks: Comparing connection strategies of innovative companies in hyperlink and social media networks

**DOI:** 10.1371/journal.pone.0283372

**Published:** 2023-03-30

**Authors:** Dorian Arifi, Bernd Resch, Jan Kinne, David Lenz

**Affiliations:** 1 Department of Geoinformatics—Z_GIS, University of Salzburg, Salzburg, Austria; 2 Center for Geographic Analysis, Harvard University, Cambridge, MA, United States of America; 3 ISTARI.AI, Mannheim, Germany; 4 Department of Economics of Innovation and Industrial Dynamics, ZEW Centre for European Economic Research, Mannheim, Germany; The University of Arizona, UNITED STATES

## Abstract

This paper seeks to unveil how (geospatial) connection strategies associated with business innovation, differ between geolocated social media and hyperlink company networks. Thereby, we provide a first step towards understanding connection strategies of innovative companies on social media platforms. For this purpose, we build a hyperlink and Twitter follower network for 11,892 companies in the information technology (IT) sector and compare them along four dimensions. First, underlying network structures were assessed. Second, we asserted information flow patterns between companies with the help of centrality measures. Third, geographic and cognitive proximities of companies were compared. Fourth, the influence of company characteristics was examined through linear and logistic regression models. This comparison revealed, that on a general level the basic connection patterns of the hyperlink and Twitter network differ. Nevertheless, the geospatial dimension (geographic proximity) and the knowledge base of a company (cognitive proximity) appear to have a similar influence on the decision to connect with other companies on Twitter and via hyperlinks. Further, the results suggest that innovative companies most likely align their connection strategies across hyperlink and Twitter networks. Thus, business innovation might effect connection strategies across online company networks in a comparable manner.

## 1 Introduction

The creation, transfer and application of knowledge are decisive factors for business innovation and thereby economic growth [1]. Accordingly, researchers and policymakers alike have long sought to unravel the fundamental dynamics which facilitate the exchange of knowledge in company networks. Traditionally, a well known path in innovation research has been to identify the key drivers of information exchange and collaboration between firms. In this regard, the predominant role of geospatial proximity between companies and the channels through which it promotes innovation have been explored in a broad range of studies [2]. One promising approach has proved to be analyzing innovation through the lens of geospatial company networks. In particular, this allows scientists to employ traditional Social Network Analysis methods (*SNA*) [3] which enable a more detailed analysis of innovation in company networks [4–6]. While some authors also specifically examine geospatial connection strategies of companies associated with more innovative behavior e.g. global buzz and local pipelines [7, 8].

Recently, similar geospatial connection patterns associated with company innovation have been observed and studied in online hyperlink company networks [7, 9, 10]. Nevertheless, it is no surprise to find these offline geospatial connection patterns in online hyperlink networks. Various studies have already attributed hyperlink networks the ability to capture real-world offline interactions between individuals or organizations [11, 12]. Specifically in recent years hyperlink networks often found usage when analyzing the spread of political ideas [13] or conspiracy theories [14]. While [15] even observed that online hyperlink communities are based on their offline geospatial locations and further emphasized their role for improving industry performance. This shows that hyperlinks offer a valuable tool for the analysis of online social interaction [16] and provide a way to study real world network interactions.

However, hyperlinks are not the only way for companies and individuals to enable interaction on the web. Social media networks such as Facebook, Instagram or Twitter, are by far the most popular tools for online social interaction nowadays. Accordingly, many researchers and organizations have recognized the potential of social media network analysis for studying and improving business innovation [17, 18]. In particular, social media can provide a competitive advantage through advanced market research [19], improve knowledge sharing [20], new ways of marketing and community and loyalty building [21, 22] or improving new product development [23]. Additionally, social media can facilitate the emergence and the distribution of knowledge flows within [24, 25] and between companies [26–28]. Hence, understanding these knowledge flows within social media networks constitutes a key factor for company innovation.

In this paper, we focus on external information and knowledge flow between companies and its observability in social media networks. Unlike previous research, we do not define challenges or opportunities of knowledge sharing [28], but rather shed light on the fundamental structural dynamics of knowledge exchange within online company networks to enable a more in depth comprehension of company innovation. In particular, our aim is to provide a profound view on how geospatial connection strategies of (innovative) companies **differ between social media and hyperlink company networks and what influence relational proximity measures and company characteristics exert**. This has the potential to reveal whether social media networks are **in theory** able to reflect connection patterns of innovative companies similar to their hyperlink counterparts. To investigate this hypothesis, we employed a novel approach which combines traditional *SNA*, innovation network analysis and social media network analysis methods. Thus, we provided a previously non-existent conjunction of these three research fields. In particular, this paper employs the methodology introduced in [9] to build a *Digital Layer* (hyperlink company network) and extends it to a social media network to build a *Digital Twitter Layer* which we call a Follower Network. This Follower Network consists of Twitter follower connections between companies and is additionally enriched with textual data from individual tweets and user descriptions. We also utilize the concept of cognitive proximity native to innovation research [29], employed it similarly to [9] for a hyperlink company network and further extended it to a social media network. To our knowledge, such a conversion of these three research fields in order to compare connection patterns in hyperlink and social media (company) networks is so far unprecedented in the literature. **To investigate to what extent (geospatial) connection strategies, related to business innovation, coincide or differ between social media and hyperlink company networks, we examine the following four research questions:**

1. What basic structural differences can be observed between social media and hyperlink company networks?
2. How do information flow patterns between companies coincide or differ across these networks?
3. To what extent do relational proximity measures differ between social media and hyperlink company networks?
4. What influence do company characteristics have on connection behavior of companies in social media and hyperlink company networks?

In order to assess structural differences, isolates, densities and clustering coefficients of both networks are compared. Information flow patterns between companies are investigated using traditional *SNA* methods such as out-degree, out-closeness and betweenness centrality. The cognitive and geographic proximity are calculated for each company according to the procedures introduced in [9] and compared across the networks. Finally, linear and logistic regression models are employed to ascertain the influence of company characteristics on centrality and relational proximity measures.

## 2 Related work

### 2.1 Innovation and geography

Innovation on a company level has been described as the process of creating and applying knowledge in order to refine products or optimize production processes [1]. In this context, the crucial influence of geography on innovation processes appears rather intuitive and has been extensively studied in the literature [2, 30]. One example of geospatial connection strategies observed with more innovative companies are global buzz and local pipelines [7, 8]. Thereby, more innovative companies entertain far reaching connections to other companies in order to exchange important knowledge, while remaining strongly embedded in local company clusters.

[29] specifically defines five relational proximity dimensions which can influence company innovation within a company network: geographic cognitive, organizational, institutional and social proximity. However, in our paper we mainly focus on analyzing and comparing geographic and cognitive proximity. This is due to the fact that organizational, institutional and social proximity are traditionally very challenging to quantify in a uniformly and comparable manner across a range of companies and industries.

Many researchers began to recognize the importance of analyzing innovation through the lens of spatial company networks. In this regard, [31] argue that company networks provide the best approach to investigate the behavior of highly innovative companies within a geospatial context. Indeed, the combination of innovation and spatial networks has allowed researchers to apply methods traditionally used in Social Network Analysis (SNA) [3]. For example, [6] explore comparative advantages and driving forces of technological innovation in China’s rare earth industry with the help of SNA methods. While [4] use SNA to investigate specifically how innovation is propagated through a network in the agricultural sector and which actors occupy positions of importance when it comes to sharing information.

### 2.2 Hyperlink networks and innovation

When investigating hyperlink networks, the question arises, to what extent these network structures are actually able to capture real world offline interactions between individuals or organizations? In this regard, many studies have shown that investigating hyperlink networks by utilizing traditional SNA procedures, as suggested in [32], can in fact provide meaningful insights into offline social interactions. For example, [11] analyze hyperlink networks with a basic *SNA* methodology to gain a better understanding of real world social interactions between NGO’s. Their findings are still today of importance as they ascertain that offline collective actions are indeed reflected in hyperlink connection behavior. In accordance with that, [12] find that hyperlink connection patterns can also reflect offline collective identity.

More recent studies build on this notion that hyperlink networks are able to capture underlying real-world phenomena. For instance, [13] investigate the transnational spread of political ideologies with the help of hyperlink networks. Likewise, researchers employ *SNA* methods to quantify structural network attributes and specify how hyperlink networks are employed to advocate political agendas [33], mobilize against certain religions [34] or how conspiracy theories develop over time on the web [14]. Furthermore, a conversion between hyperlink analysis and social media is provided by [35] who investigate how online connections among Korean politicians have developed from the Web 1.0 to the Web 2.0. However, their remarkable idea was not only to compare hyperlink connections of politicians websites over time, but further to contrast hyperlink networks with politicians activity on the social media microblogging service Twitter. In this regard, they find that the hyperlink and Twitter networks of politicians differ considerably. In particular, the Twitter network is more densely connected than the hyperlink network. Our paper took inspiration from their approach and compared hyperlink and Twitter networks. However, their idea was extended to a larger company network, while specifically focusing on the comparison of (geospatial) connection strategies associated with innovation and knowledge generation. In recent times, [36, 37] provide examples of the conversion of hyperlink and social media network analysis. [36] investigate the visibility of media and social media platforms during the formation of the Spanish government and which types of media are prominent in the hyperlink connections of Twitter posts. [37] on the other hand examine the exposure diversity of users to news. As becomes clear from the latest literature converging hyperlink and social media network analyses, there remain still a lot of potential pathways, especially with regards to comparing both network structures in an economical and innovation context.

Hyperlink networks have also been used in the context of analyzing urban places [38] and economical analysis of the tourism industry in Australia [15]. Both studies are particularly interesting as they associate hyperlink activities indistinctly with the geospatial dimension. [15] even emphasizes that the observed community formation reflects the geospatial location and that education about the potential of hyperlink connections could increase industry performance. This is remarkable as they point out how structural components in form of a broader connectedness via hyperlinks can influence economic performance.

In this regard, [9] observe that connection patterns of innovative companies in geolocated hyperlink networks reflect those of innovative companies in offline company networks. Therefore, they use a so-called Digital Layer, which is in principle a geolocated company hyperlink network enriched with the textual contents of the corresponding company websites. This allows the Digital Layer to assess relational proximity measures such as geographic proximity and cognitive proximity, which are traditionally used to ascertain the innovation potential of a company within a network [29]. Therefore, the Digital Layer offers advanced contextualization and interpretation opportunities for company hyperlink connections with regards to innovation. The findings of [9] reveal that innovative companies connect in significantly different patterns among each other via hyperlinks than their non-innovative counterparts. In particular, they observe patterns which are often found in offline knowledge generation processes [7, 8]. [10] have since investigated what specific connection patterns in hyperlink company networks are directly related to company innovation.

### 2.3 Social media networks and innovation

Plenty of research has highlighted many different ways through which social media can influence company innovation and performance [17]. While social media has proved to be useful for various purposes such as market research [19], new product development [23], marketing or community building [21, 22], it shines through its opportunity for knowledge exchange [20]. In this regard, more recent findings specifically emphasize the possible mediator role of social media for enhancing the ability to capture and process knowledge important for company innovation.

While the the process of sharing and absorbing knowledge can take place on an internal [24, 25] and external company level [26–28], the correct way of knowledge management constitutes a key factor for company innovation [39]. Note, in this paper the main interest lies in external knowledge management. One promising approach among many is in this context to use social media platforms to directly gather consumer knowledge, ideas and needs to optimize so-called open innovation processes [26, 40].

Nevertheless, extant literature has not yet assessed the exact external patterns in which knowledge flows from company to company in social media networks. Specifically, the question whether knowledge flows through social media networks similarly as through hyperlink or offline company networks still remains unanswered. We examine this in the course of this paper.

## 3 Data

**Fig 1:**
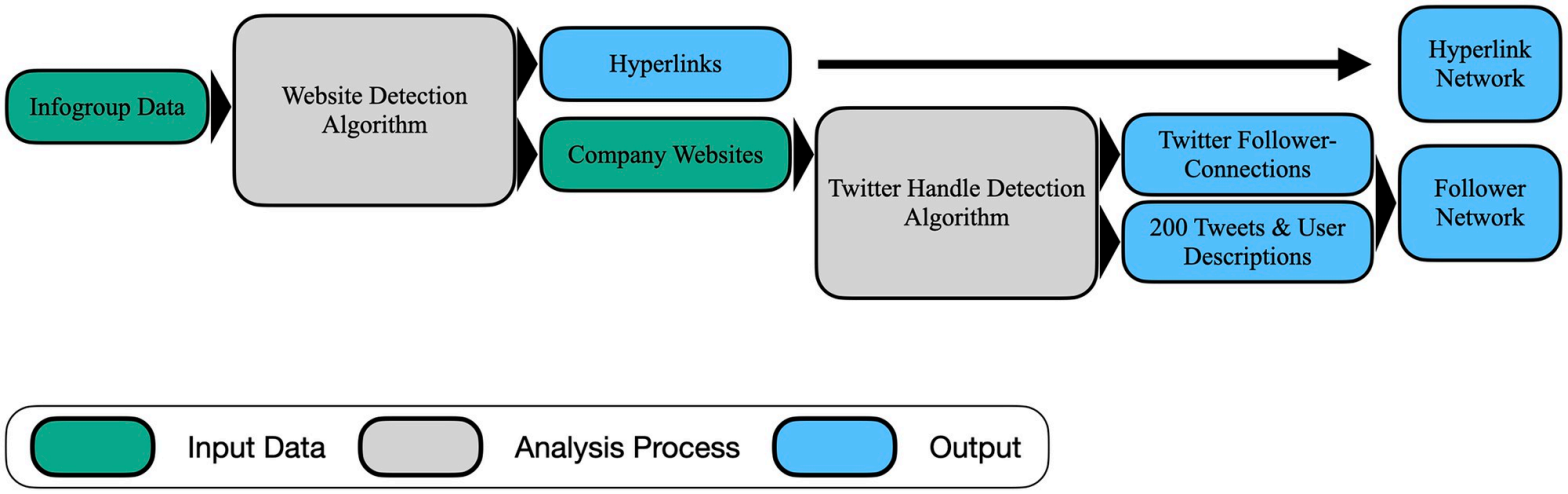
Data acquisition and preprocessing.

Two main data sources were employed in this work. First, the *Infogroup Business Data 2017* data set, obtained from the Harvard Dataverse, was used to create search strings, obtain company characteristics and identify company websites. With these company websites and the corresponding hyperlink connections between them, a so called Hyperlink Network was build. Second, Twitter handles were extracted from company websites to construct a geolocated Twitter company network comprised of inter-company Twitter follower connections (Follower Network). Fig 1 illustrates the data acquisition and analysis steps which were necessary to build the Hyperlink and Follower Network. The following sections explain the whole data collection and analysis process in further detail.

### 3.1 Website Detection Algorithm

The list below specifies the algorithm with which the **company websites’s** were collected. As a starting point, the Infogroup Business Data 2017 was utilized, which provides 14.8 million observations for companies located in the United States of America (U.S.).

- **Exclusion of professionals:** Professionals (e.g. doctors or attorneys) have on average fewer employees than companies (mean 2.3 compared to a mean of 13.6). The analysis of similar circumstances in earlier work on the German *Digital Layer* [9], revealed that the probability of a business having a website decreases significantly with its size (number of employees). Hence, 2, 643, 002 professionals were excluded from the further analysis in order to optimize the time over reward ratio of the website scrapping algorithm.
- **Exclusion of small business:** According to the same rationale as described above, also all companies with fewer than 10 employees were excluded.
- **Exclusion of Branches:** Locations classified as company Branches were excluded as well. This decision is again based on prior experience from the German *Digital Layer* [9], where Branch type locations often shared their websites with other locations from the same Branch. E.g. all restaurant locations of a fast food chain have the same Branch-website on which, information on single locations can be found. Hence, including all Branch type locations would only flood the data set with duplicates.

After the prior three steps the data set contained 1,870,588 observations. Next we conducted a website search according to the following procedure:

- **Search string:** For each company its name, city and state were extracted from the *Infogroup Business Data 2017* data set and concatenated into a search string. This search string was then randomly fed into one of the following search engines: Google, Bing, Yahoo!, Duckduckgo, Startpage, Aol, Dogpile, Ask or Mojeek. Subsequently, the first ten websites were further processed.
- **Excluding too frequently occurring websites:** All websites which were encountered more than 20 times in the whole search process (threshold again based on prior experience from [9]) were afterwards excluded in order to filter out social media websites or other company information websites, for example Facebook, LinkedIn or Crunchbase.
- **Website validation:** For each of the ten websites obtained per search string, the textual content of 25 sub-webpages was scraped. Subsequently, it was examined on how many sub-webpages, the name of the company and its address could be found. If 90% or more subpages contained the company name and address, the found domain was considered a correct match (all thresholds are based on prior experience from [9]). Therefore, a so-called decision score was calculated, indicating what percentage of the sub-webpages included at least the name or the corresponding company address. The decision score could be calculated for 1,020,496 companies.
- **Hyperlink and Twitter handle extraction:** Finally, all hyperlinks to other companies’ websites were extracted for all retrieved websites (*Website Detection Algorithm*). Company Twitter accounts were also extracted via Twitter plugins on the found websites (*Twitter Handle Detection Algorithm*).
- **Focusing on the IT sector:** After focusing on the IT sector, 112,653 companies remained in the sample. Since, IT focused companies tend to be more technology-savvy, they are more likely to have websites and Twitter accounts. Therefore, narrowing the analysis to the IT sector only might provide more complete data and thereby allow for a more robust interpretation of the results. However, this needs to be considered when interpreting the results.

In the Hyperlink Network single nodes denote company websites, while edges reflect hyperlink connections between company websites. While, the textual contents of the 25 sub-webpages are used to calculate the cognitive proximity of a company. Note that hyperlinks between company websites may change over time. Therefore, the hyperlinks investigated in this study have been retrieved in the time span from the 01.10.2020 to the 01.11.2020.

### 3.2 Twitter Handle Detection Algorithm

As explained above, company Twitter handles were extracted from the company websites via plugins. Furthermore, for every company their follower connections, their user descriptions, and the corresponding 200 last tweets were collected. In the Follower Network, Twitter accounts represent nodes and the collected Twitter follower connections represent the edges. The tweets and user descriptions are merged together and used as a comparative textual measure (cognitive proximity).

Ultimately, the attributes of interest were only successfully retrieved for 11,892 companies. This is mainly due to the fact that only a fraction of the companies had a unique and detectable Twitter handle, while still having a decision score higher zero. The Twitter follower connections were collected in the period from the 11.10.2020 to the 22.11.2020. The last 200 tweets for each company and its user description on Twitter, were collected in the period from the 24.11.2020 to the 28.11.2020.

Finally, since the companies Facebook and Twitter are linked to almost every company website or Twitter account, they were excluded from the analysis. As they represent strong outliers and mitigate the effectiveness of statistical methods.

## 4 Methods

**Fig 2:**
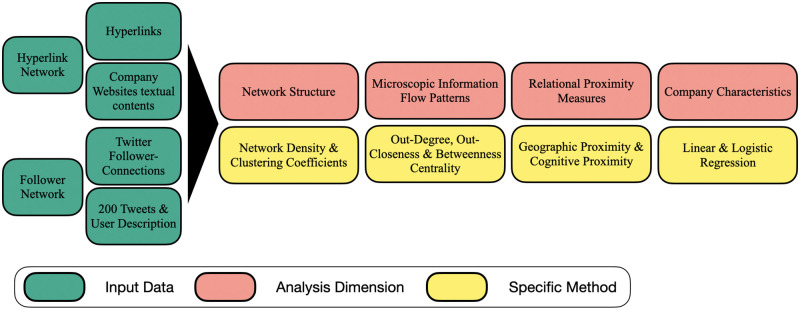
Analysis dimensions and specific methods.

In order to ascertain differences of companies’ connection strategies across the Hyperlink and Follower Network, both networks are compared along four dimensions. The specific approach is represented in Fig 2.

### 4.1 Structural features

In order to provide a basic understanding of the structural features of the Hyperlink and Follower Network, traditional methods for the analysis of social networks are used [3]. Many studies comparing hyperlink structures have examined e.g. network densities [5, 11, 35] as well as clustering coefficients of hyperlink networks [5, 11]. In this specific case, the density allows to compare how actively companies follow each other on Twitter or connect via hyperlinks. The clustering coefficient, on the other hand, captures the overall connectedness of the networks. In particular, the clustering coefficient of a node determines whether the neighbors of a random node are strongly connected or whether many nodes simply follow single major players in the network [41].

### 4.2 Information flow patterns between companies

Further, the out-degree, out-closeness and betweenness centrality are utilized to ascertain fundamental differences in information flow patterns between companies in the Hyperlink and Follower Network. In general, centrality measures such as degree, betweenness or closeness are common tools to identify the influence of an individual actor on the information flow within a network [42]. As a results they have been widely used in the context of innovation analysis in spatial company networks [5].

In particular, the underlying rationale is that companies with a high degree centrality can directly influence more companies through active or passive information and knowledge dissemination. However, the final comparison will focus on out-degree centrality, since this measure is more convenient in capturing the active decision of a company and thereby actor-level interaction. Similarly, the out-closeness centrality reflects the ability of a company to reach all others and thereby spread information faster within a network. While, the betweenness centrality of a company captures how much influence it has on the information flow between other companies within the network [42].

### 4.3 Relational proximity measures

In general, researchers have defined relational proximity measures which model the influence of certain external and internal properties of a company on its innovation potential [29]. In this regard, [9] have shown that it is in fact possible to calculate meaningful versions of these relational proximity measures in company hyperlink networks. Hence, this research leans on their idea to calculate relational proximity measures for a hyperlink company network and further extends their approach to a Twitter follower network. In particular, geographic and cognitive proximity are calculated and compared for both networks.

#### 4.3.1 Geographic proximity

The geographic proximity captures the average geographic distance of one company to all its directly linked neighbors [9]. Therefore, it reveals whether edges between companies span on average over the same distances across the Hyperlink and Follower Network. The geographic distance was calculated using the haversine formula and the exact company headquarter coordinates. Subsequently, for each company all its edge distances were summed and divided by its total number of edges. Note that assuming that the weights of the networks correspond to the geographic distance between companies, this definition of geographic proximity is no novelty, but very similar to the weighted degree introduced by [43].

#### 4.3.2 Cognitive proximity

The cognitive proximity aims at ascertaining similarities between the knowledge bases of companies based on their textual data provided on their websites (Hyperlink Network) or through their last 200 tweets and user description on Twitter (Follower Network). In fact, [9] have already explored the knowledge base of a company via its website’s textual contents. Beyond that, companies use social media platforms such as Twitter to present themselves to costumers and increase their business value [44]. Put differently, companies use industry and product specific language on social media to stand out which suggests that conclusions on their knowledge base could be inferred from their social media posts.

In order to determine similarities between textual contents of Tweets and websites, a term frequency-inverse document frequency (tf-idf) algorithm is employed [45]. In general, this algorithm transforms each document into a high-dimensional vector space in which differences can be calculated. More specifically, the idea is to set the frequency with which a word occurs within a given document in relation to how often this word appears within a given dictionary. This dictionary of size n is usually composed of all words found in all documents under consideration, i.e. the entire text corpus. Hence, each document can be represented as a sparse n-dimensional vector. Similarities between these vectors are then calculated separately for the Hyperlink and the Follower Network using the cosine similarity. This is a common approach in natural language processing [46, 47]. Further, according to the so-called popularity based filtering, the dictionary is restricted to words with a document frequency of at least 1.5% and at most 65%. This procedure filters out words which do not provide useful information on the document’s topic.

In the case of the Hyperlink Network, the text on an individual company’s website and all sub-websites is perceived as a single document and the sum over all websites forms the dictionary. In contrast, in the Follower Network a single document is defined as the conglomerate of all available last 200 tweets of an individual company and its user description. Again, the corpus is the sum over all documents. Note, since this is a novel approach, there is no common benchmark on how many tweets to use. Hence, a sample size of 200 was chosen to optimize the scraping process and the calculations of the cognitive proximity. Additionally, the cognitive proximities did not change considerably for larger tweet sample sizes.

### 4.4 Influence of company characteristics

Studies like [48, 49] clearly advocate the positive relation between innovation and company characteristics such as e.g. company size. Assuming that a company’s innovativeness influences its connection behavior, it seems reasonable to investigate whether company characteristics might influence connection patterns in the Hyperlink and Follower Network differently. Therefore, we first calculate linear regression models for the Hyperlink and Follower Network. Those models explain variations in centrality measures and in relational proximity measures through company characteristics such as sales per annum (sales), number of employees (size) and year of establishment (year).

**Fig 3:**
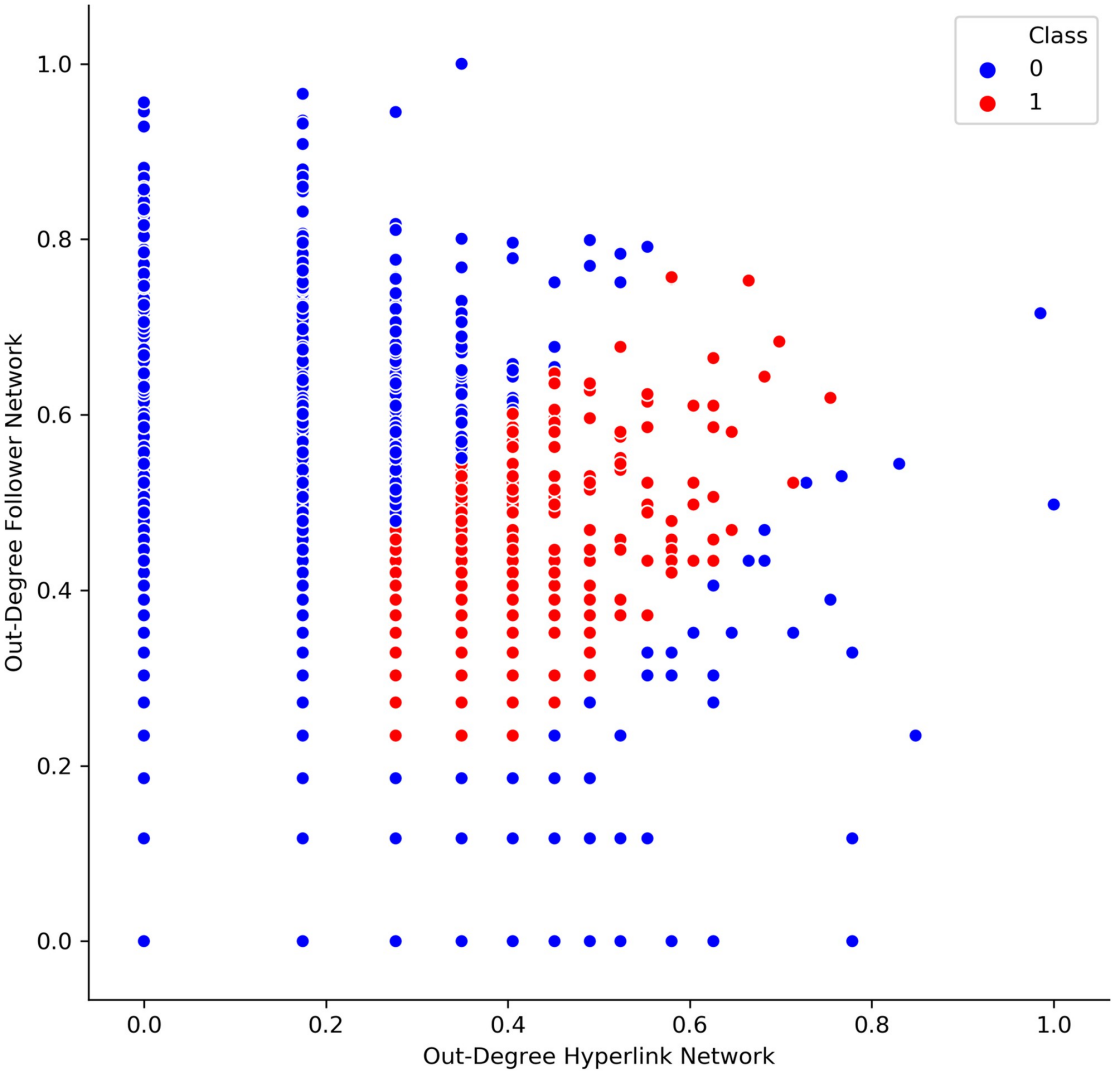
Categorizing data with the help of sliding windows.

Second, a logistic regression approach is used to determine the (logarithmic) chance of a company to achieve high values of centrality or proximity measures across both networks explained by the underlying company characteristics. Thereby, the dependent variables are categorical variables that distinguish whether a company has high values (given a certain tolerance window) in both networks (category 1) or not (category 0). These tolerance windows slide over the data and thereby create a corridor around the axes bisecting line. Fig 3 illustrates such a corridor for the out-degree scatter plot. While, red points indicate that a company is in category 1 and blue points indicate that a company falls into category 0.

This sliding tolerance window begins at a certain threshold. This is supposed to guarantee that mainly companies which have relatively high values of the investigated centrality or proximity measures (key players), fall into category 1. However, the greatest vulnerability of this approach is that there are no general guidelines on whether companies centrality or proximity measures coincide sufficiently. Therefore, two different combinations of sliding windows and starting points were utilized. First, for relatively balanced scatter plots (e.g. Fig 3) a sliding window size of 0.2 and a starting point of 0.2 were employed. Second, for data samples with major outliers and less companies with equally high values in both networks, a sliding window of size 0.3 and a starting point of 0.01 was chosen. Thereby, the sliding windows and starting points were set to optimize the convergence of the logistic regression algorithm. This approach is highly debatable and should therefore not be understood as a perfectly optimized method (cf. Discussion).

## 5 Results

### 5.1 Performance of the *Website* and the *Twitter Handle Detection Algorithms*

#### 5.1.1 *Recall* and *Precision*

First, in order to ascertain the performance of the *Website* and the *Twitter Handle Detection Algorithms* a random sample of size 200 was drawn from the 112,653 companies from the IT sector. Table 1 shows the *Recall*, *Precision* and *F1-score* for the *Website* and the *Twitter Handle Detection Algorithms*, evaluated on the basis of these 200 companies.

**Table 1:** *Recall*, *Precision* and *F1-score* of the *Website* and *Twitter Handle Detection Algorithm* based on a sample of 200 companies from the 112,653 companies in the IT sector.

Detection algorithm	Recall	Precision	F1-score
**Websites**	61.70%	90.63%	73.42%
**Twitter handles**	35.56%	100%	52.46%

The *F1-score* is relatively low for both algorithms (52.46% Twitter handle Detection a 73.42% *Website Detection Algorithm*). Further, comparably low *Recall* values were observed, indicating that 61.70% of all existing websites and only 35.56% of all existing Twitter handles were found. Furthermore, for the 11,892 companies which were kept for the final analysis, a sufficiently good *Precision* of 97% was estimated with the help of an additional random sample of size 100.

#### 5.1.2 Influence of company characteristics

Second, the sensitivity of the *Website* and *Twitter Handle Detection Algorithms* to company characteristics such as year, size and sales, was examined. In particular, the following tests were utilized to compare the multivariate non-parametric distributions, as suggested by [50] and as implemented in the *R* package *npmv*:

- ANOVA type test
- McKeon approximation for the Lawley Hotelling Test
- Muller approximation for the Bartlett-Nanda-Pillai Test
- Wilks Lambda

Their results indicate conclusively that no significant structural differences in company characteristics (sales, size and year) can be attested, between companies with only a detected website or those with a detected Twitter handle.

Additionally the same statistical tests did not yield any significant differences in company characteristics between companies which were detected by the algorithms and those which were not. Put differently, the company structure (size, sales and year) does not differ significantly between companies for which a website was **detected** compared to all companies with **detectable** websites but for which no website was detected. The same holds true for Twitter handles.

#### 5.1.3 Influence of connections

Third, the number of connections made by companies which the algorithms detected and those which were not detected were compared. For this purpose, a Welch approximation t-test was utilized. It did not confirm significant differences in the number of hyperlink connections made by companies for which a website was detected and those for which no website was detected even though it would be detectable. **However, those companies for which the algorithm detected a Twitter handle did have significantly more follower connections than those for which no Twitter handle was detected even though it would be detectable**. The discussion will exemplify possible ramifications of this finding in more detail.

### 5.2 Structural differences between the Hyperlink and Follower Network

Table 2 presents the main structural features of the Hyperlink and Follower Network. In particular, it shows the number of edges, the in-/out-degree, the maximal degree, the number of isolates, the density and the clustering coefficients of the Follower and Hyperlink Network. To be specific, the Hyperlink Network has 5,670 edge-connections, while the Follower Network has 117,396. This corresponds to almost 21 times as many connections in the Follower Network. Accordingly, a similar difference is observable for average in-/out-degree centrality of the Hyperlink (0.4769) and Follower Network (9.8735). Further, the highest maximum number of connections observed are 429 in the Hyperlink Network and 1,382 in the Follower Network. This difference is only about 3 times larger. Additionally, 7,908 companies (66,51%) in the Hyperlink Network and 2,006 companies (16.87%) in the Follower Network do not entertain any connections (Isolates). In the Hyperlink Network the density (0.00004011) indicates that 0.004011% of all possible connections are made. In comparison, in the Follower Network the density is again approximately 21 times higher, with 0.00083047. Moreover, the average clustering coefficient in the Follower Network (0.09522137) is approximately 30 times higher than in the Hyperlink Network (0.00325684). Since the clustering coefficients are averages over a distribution of values, differences between these distributions were reaffirmed with the help of a paired t-test.

**Table 2:** Structural differences between the Hyperlink and Follower Network.

Network structure	Hyperlink Network	Follower Network
**Edges**	5,670	117,396
**In-/out-degree**	0.4769	9.8735
**Max degree**	429	1,382
**Isolates**	7,908	2,006
**Density**	0.00004011	0.00083047
**Clustering coefficient**	0.00325684	0.09522137

### 5.3 Information flow patterns between companies

**Fig 4:**
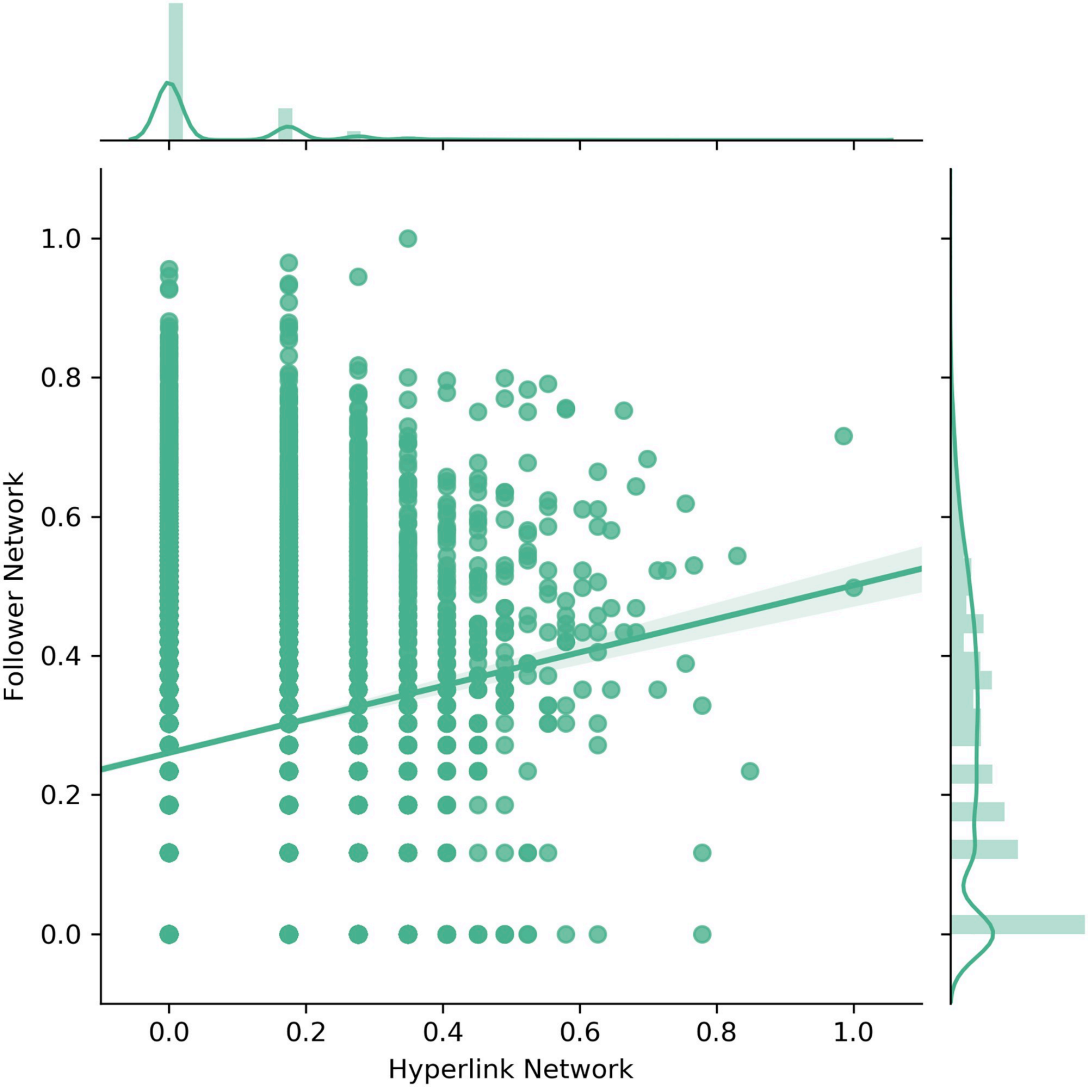
Scatter and density plots for degree centrality.

**Fig 5:**
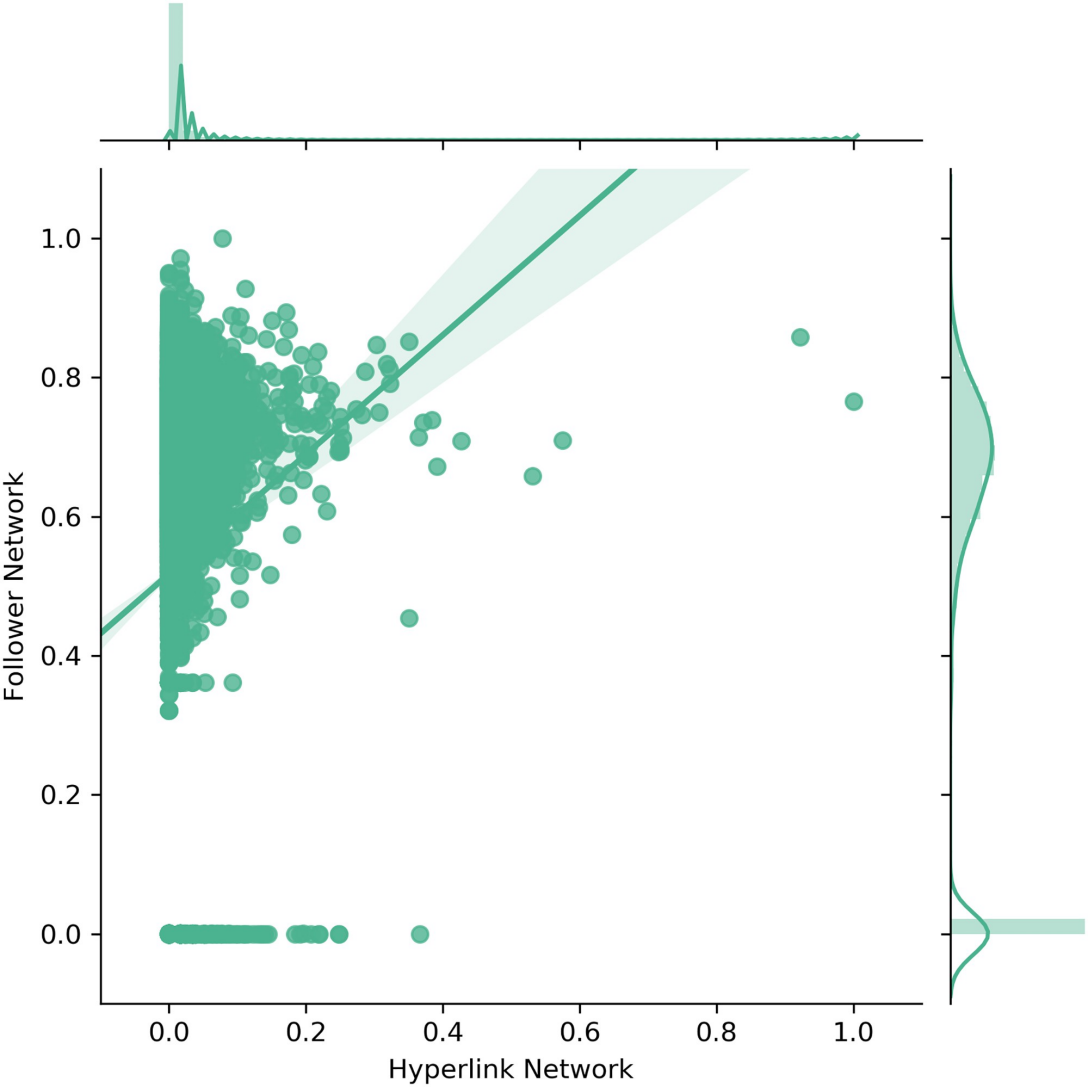
Scatter and density plots for closeness centrality.

**Fig 6:**
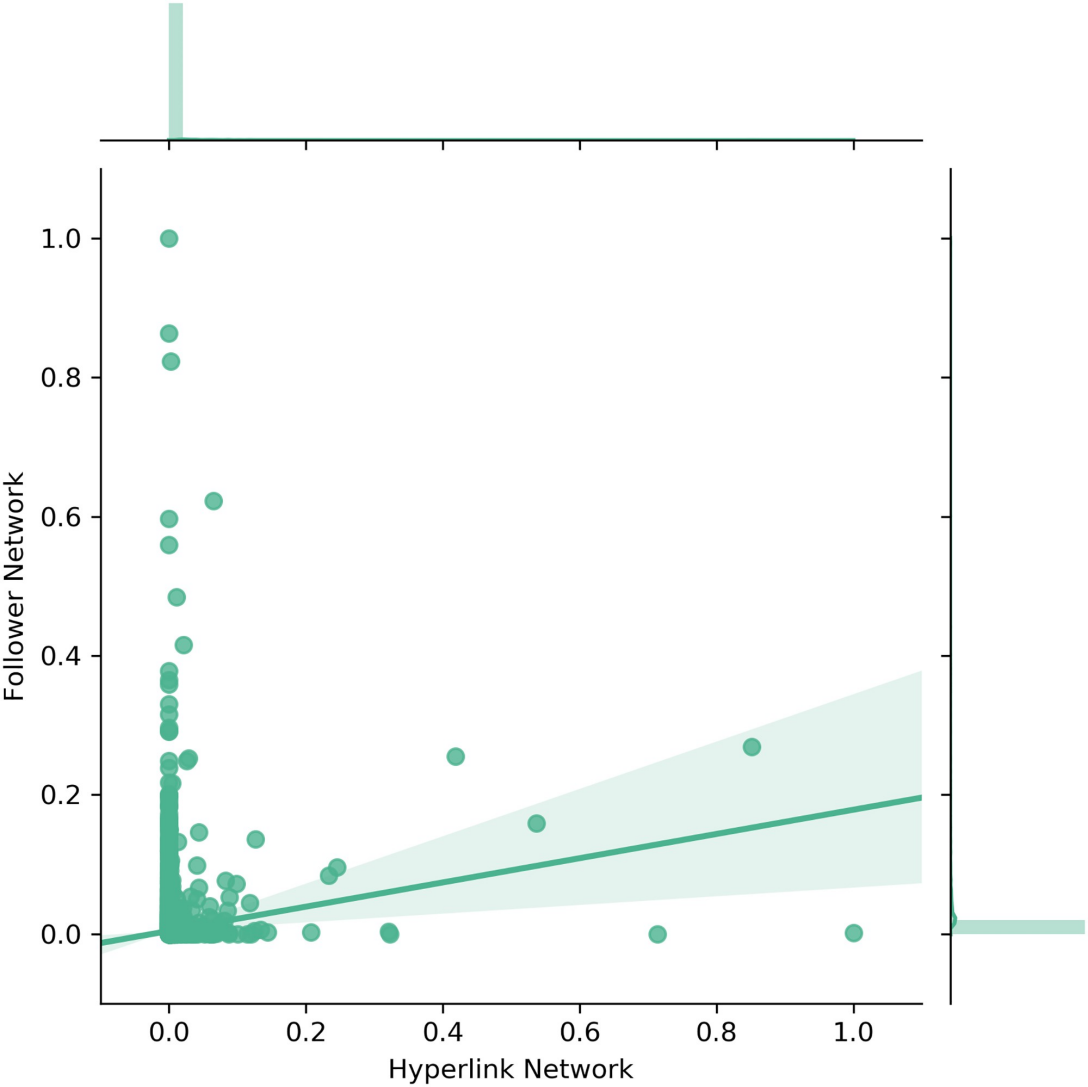
Scatter and density plots for betweenness centrality.

The subsequent figures show the scatter plots as well as the corresponding densities for the out-degree (Fig 4), the out-closeness (Fig 5) and the betweenness centrality (Fig 6). The figures include linear regression lines which indicate how the observed values in the Follower Network (y-axis) are explainable through the values in the Hyperlink Network (x-axis). Beyond that, the values in both networks were separately log-transformed and normalized.

Note, the large sample size might distort the results when comparing the distributions with traditional statistical tests. Since a statistical test will almost always find significant differences in a sufficiently large sample (about 10,000 or more), even if the true distribution difference is negligible [51]. This ascribes additional importance to the visual comparison of the distributions. Furthermore, we are not only concerned with establishing whether differences between the two distributions are the subject of pure chance or not (statistical significance), but rather what the actual effect size difference [51] is. Thus, effect size differences, which are not as susceptible to the sample size [51, 52], were calculated according to [52]. However, to keep this analysis concise, effect size differences were only specifically mentioned when they provided further insights not directly derivable from the visual analysis and the standart statistical tests used (Kolmogorow-Smirnow test for parametric and Wilcoxon matched-pairs signed-ranks test for non-parametric distributions).

Additionally, the major structural difference between the Hyperlink and Follower Network lies in the amount of edge-connections, as shown in Table 2. Hence, in certain cases it might be reasonable to restrict the analysis to only those companies which do entertain connections in both networks (non-zero companies). This restriction allows to more accurately assess differences in connection behavior between networks, which would otherwise be overshadowed through the large edge connection difference.

Fig 4 shows that the out-degree centrality distributions do differ considerably between the Hyperlink and the Follower Network. Accordingly, statistical tests did not attest any similarities between the out-degree distributions of the two networks. However, the scatter plot indicates that at least a few companies have equally high values in both networks.

The distributions of the out-closeness centrality (Fig 5) vary even stronger between the Hyperlink and Follower Network. Again, statistical tests rejected similarities between the distributions. However, the scatter plot shows that companies in the Follower Network which have an out-closeness centrality close to the mean of the normal distribution (at around 0.7), also tend to have higher out-closeness in the Hyperlink Network.

At first glance, the betweenness distributions in both networks (Fig 6) appear to coincide more than those of out-closeness and out-degree centrality. This is comes as a result of the fact that both distributions are characterized through a high spike close to zero. Again statistical tests did not attest any similarities between the networks. However, clearly similarities are visible between the distributions. This is reaffirmed by the fact that the effect size difference between the means of the betweenness centrality distributions is small (Cohen’s d: −0.3540).

### 5.4 Relational proximity measures

**Fig 7:**
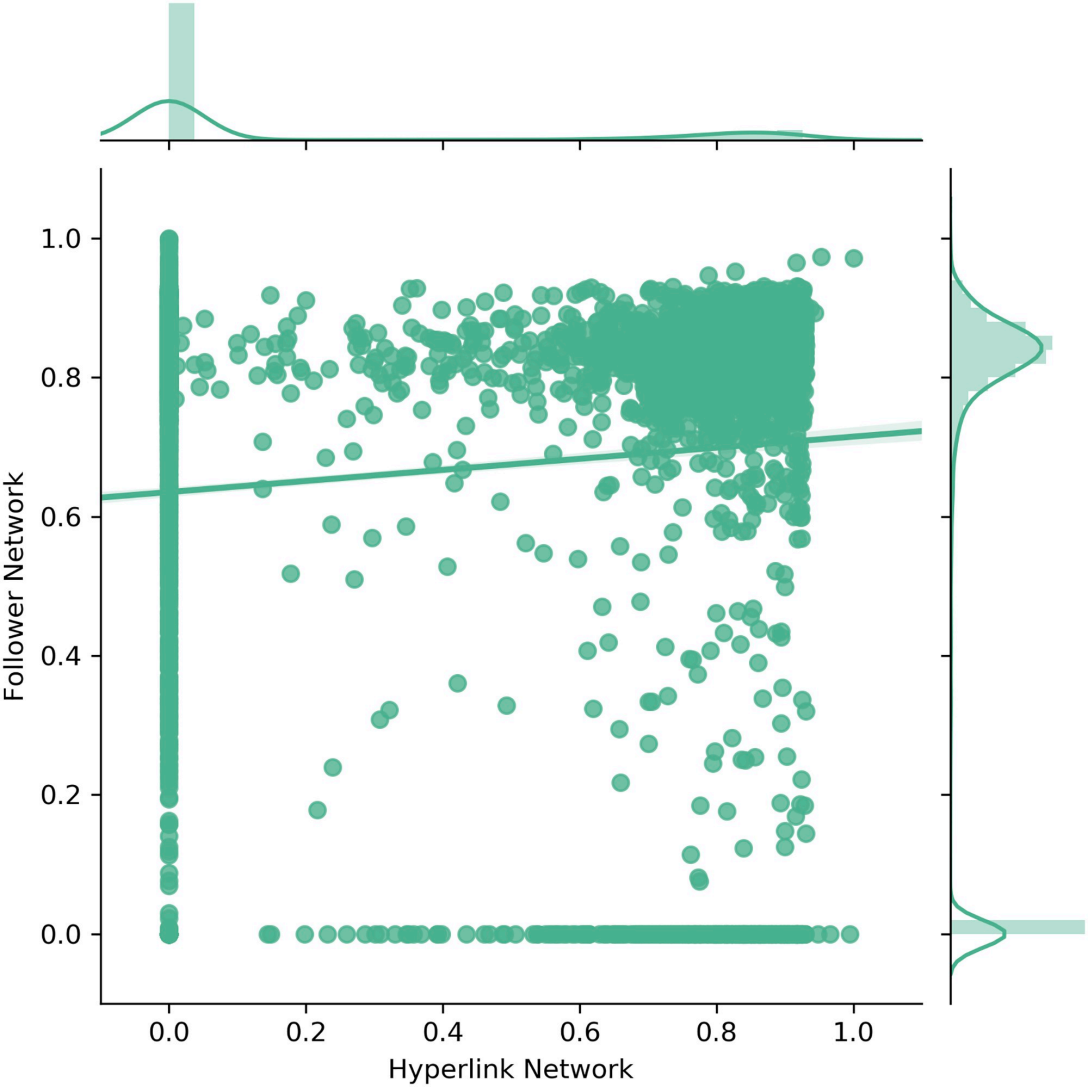
Scatter and density plots for geographic proximity.

**Fig 8:**
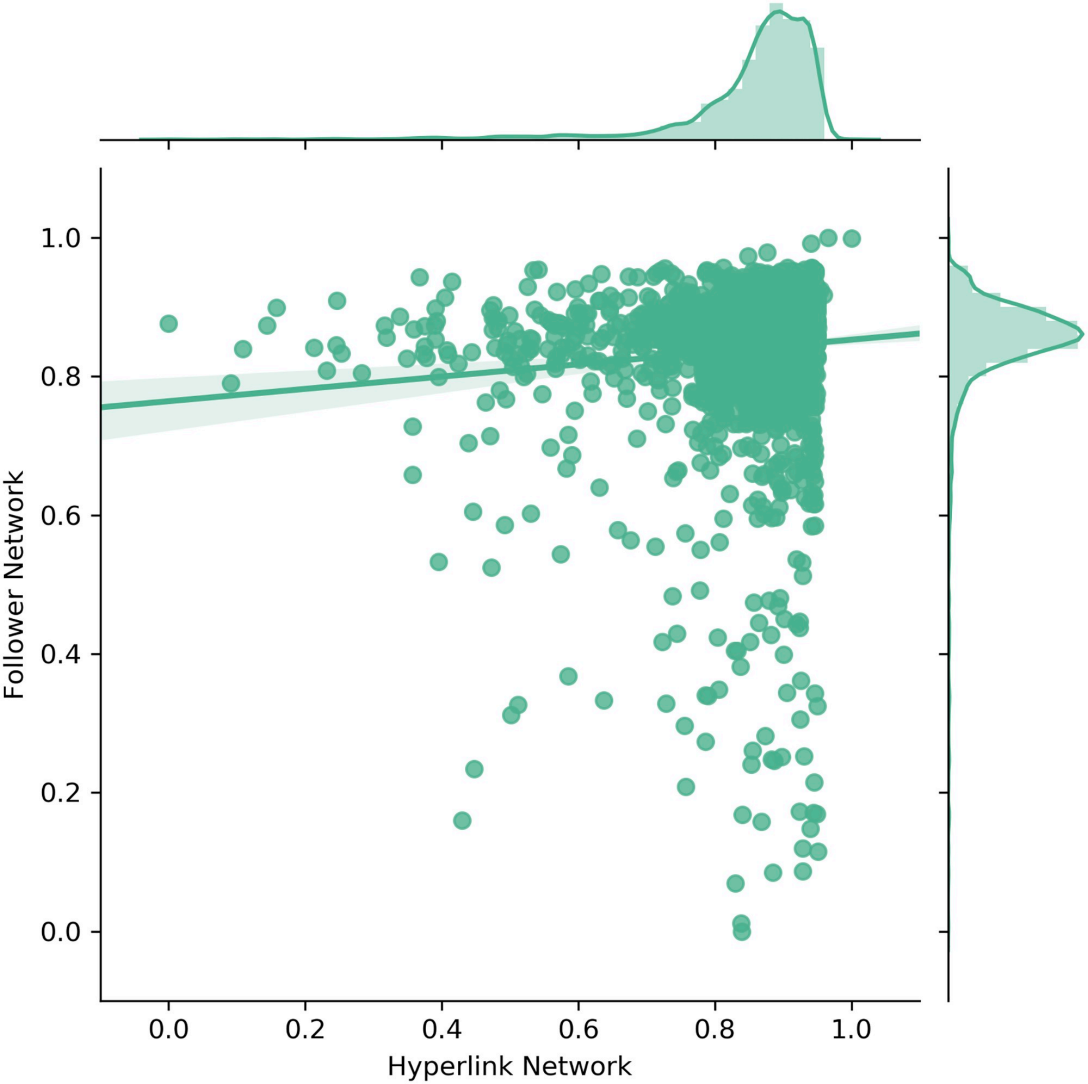
Scatter and density plots for geographic proximity non-zero companies.

**Fig 9:**
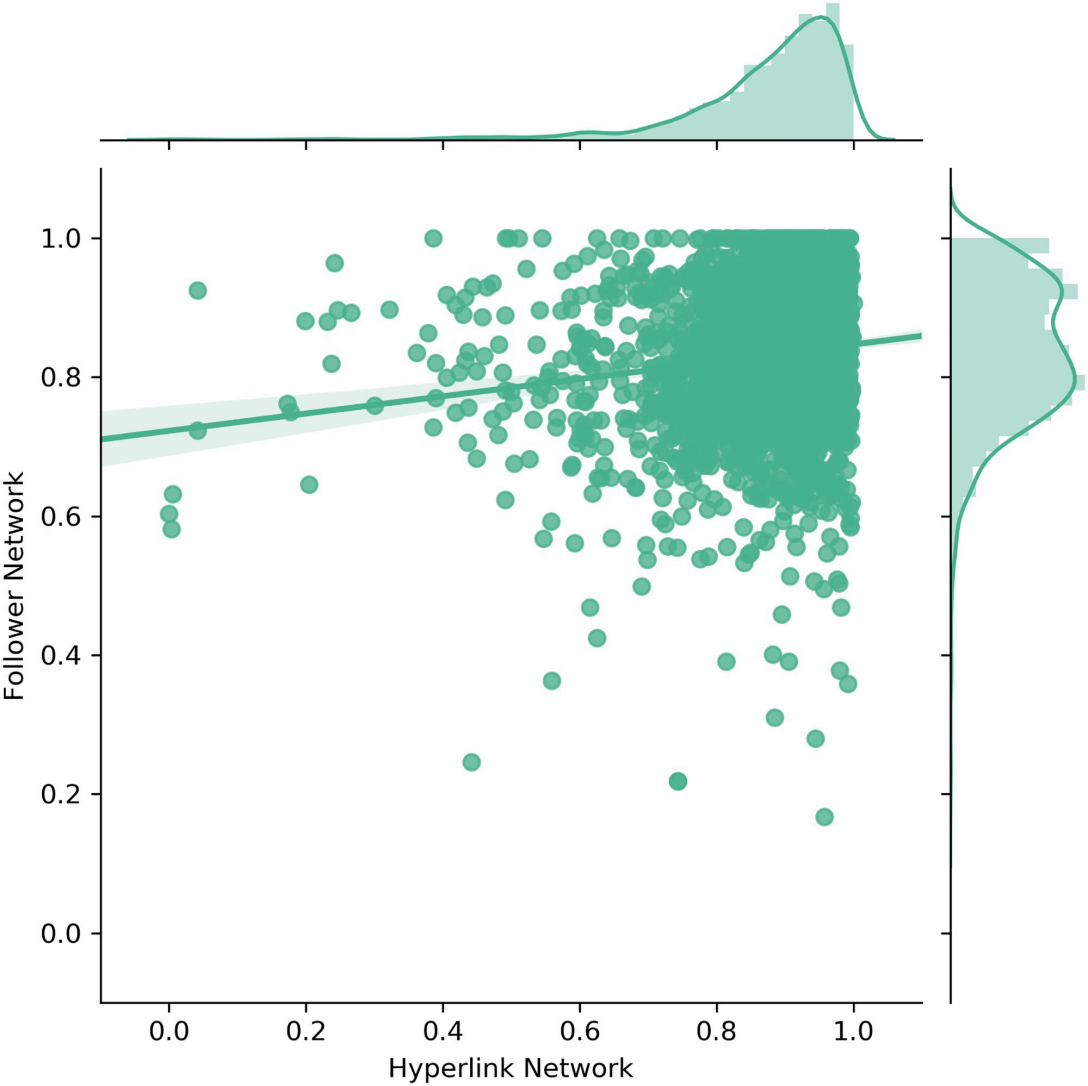
Scatter and density plots for cognitive proximity.

Next, the scatter and density plots for the geographic proximity (Fig 7), the geographic proximity of non-zero companies (without zero distances) (Fig 8) and the cognitive proximity (Fig 9) are discussed in a similar fashion as before.

The scatter plot of the geographic proximity (Fig 7) shows some similarities. However, the distributions reveal clear differences, which are mainly driven by the fact that a majority of companies in the Hyperlink Network have a geographic proximity of zero, indicating no edge connections to other companies. Again, statistical tests rejected the hypothesis that both samples originated from the same distribution. Nevertheless, the scatter plot clearly reveals that many companies tend to have equally high geographic proximities in both networks. This effect appears to be amplified for higher values of geographic proximity. Hence, it might be reasonable to focus on non-zero companies, to further investigate this phenomenon.

Fig 8 depicts the geographic proximity of all non-zero companies i.e. companies that have at least a geographic proximity larger zero in both networks (in total 2,643). Now, in both the Hyperlink and Follower Network the majority of companies are located within a skewed peak at the higher end of the scale. Nevertheless, statistical tests again reject the hypothesis that the differences between the distributions are the result of mere chance. However, the actual effect size difference between the distribution means is only very small (Cohen’s d: 0.0939). This result in combination with the clearly observable similarity in the scatter and density plots confirms that the distributions of geographic proximity for non-zero companies coincide to a certain degree across the Hyperlink and Follower Network.

Moreover, in Fig 8 there is a clear tendency visible in the data. Namely, the higher the geographic proximity of a company in one network, the more likely it seems to have a similarly high geographic proximity in the other network. This observation was also confirmed through a logistic regression model. In particular, the geographic proximity values in the Hyperlink or Follower Network, both had a positive significant effect on the chance of a company having equally high geographic proximity in both networks. Equal geographic proximity was defined using a sliding window with size and starting point of 0.2.

Similarly, Fig 9 shows the cognitive proximity of non-zero companies (in total 2919 companies). Adjusting the scope to these non-zero companies is again motivated by the fact that the large amount of companies with zero cognitive proximity render the distribution comparison less expressive. In particular, Fig 9 reveals that the cognitive proximity of non-zero companies in both networks appears to be relatively high and comparable. Nevertheless, statistical tests did not confirm any similarities between the two distributions. However, testing for effect size differences revealed only a small significant mean difference (Cohen’s d: 0.3104). Again, this result in combination with the scatter and density plots confirms that the cognitive proximity of non-zero companies is distributed relatively similarly across the Hyperlink and Follower Network.

### 5.5 Influence of company characteristics

Table 3 presents the results of the linear regression models which measure the influence of company characteristics on centrality and proximity measures in both networks. All standard errors account for heteroscedasticity. Moreover, it should be noted that the number of observations per regression model is significantly lower than the utilized 11,892 companies in the Hyperlink and Follower Network. This is due to missing values in company characteristics.

**Table 3:** Influence of company characteristics in the Hyperlink and Follower Network.

Dependent variable	Network	constant	log(sales)	log(size)	year	R^2^ adj.	Obs.
**Out-degree**	Hyperlink	0.8384	0.0047	**0.0154** **	-0.0003	0.0018	4,886
	Follower	-1.9080	**-0.0187** *	**0.2832** ***	0.0015	0.0546	4,886
**Out-closeness**	Hyperlink	0.0171	0.0001	**0.0013** **	-0.0000	0.0012	4,886
	Follower	-0.2837	-0.0012	**0.0517** ***	0.0004	0.0351	4,886
**Betweenness**	Hyperlink	-0.0116	-0.0006	0.0013	0.0000	0.0046	4,886
	Follower	0.0121	**-0.0011** ***	**0.0034** ***	-0.0000	0,0083	4,886
**Geographic proximity**	Hyperlink	-612.1089	6.4301	16.8790	-0.5344	0.0006	4,886
	Follower	-2,267.5045	-5.4726	**91.4071** ***	**1.8377** ***	0.0081	4,886
**Cognitive proximity**	Hyperlink	0.4560	**0.0040** **	0.0033	-0.0002	0.0007	4,886
	Follower	-0.0420	0.0005	**0.0304** ***	0.0002	0.0168	4,886

Note:

* p<0.1;

** p<0.05;

*** p<0.01

The results show that for the depending variables out-degree and out-closeness centrality, the coefficient size of a company has a consistently positive significant effect in both networks. However, this effect is is more pronounced in the Follower Network without exception. In the Hyperlink Network no significant effects on betweenness centrality were observed. While in the Follower Network the sales and size of a company have a significant and negative and positive influence on betweenness centrality. For geographic proximity, the regression models found significant and positive coefficients for size and year in the Follower Network and no significant coefficients in the Hyperlink Network. Furthermore, cognitive proximity in the Hyperlink Network is only significantly influenced by the coefficient sales. While in the Follower Network only the company size has a significant positive influence on cognitive proximity.

Table 4 shows the results of the logistic regression model and the effect company characteristics such as sales, size and year have on the (log) chance of a company to have equally high values with regards to centrality and proximity measures, across both networks. Again, all standard errors account for heteroscedasticity. In particular, the results reveal that the size of the company has a significant influence on the logarithmic chance of a company to have equally high values across the Hyperlink and Follower Network of out-degree, betweenness centrality and geographic and cognitive proximity. Similarly, the logarithmic chance to have equally high out-closeness in both networks increases with higher sales values of a company.

**Table 4:** Influence of company characteristics on similar connection strategies in the Hyperlink and Follower Network.

Dependent variable	constant	log(sales)	log(size)	year	AIC	Obs.	SW size	SW start
**Out-degree**	9.1901	-0.0014	**0.2545** ***	0.0027	1,542.118	4,886	0.2	0.2
**Out-closeness**	-22.9935	**0.1482** ***	0.0170	0.0075	86.458	4,886	0.3	0.01
**Betweenness**	-3.9066	-0.0807	**0.5038** ***	-0.0012	237.851	4,886	0.3	0.01
**Geographic proximity**	0.1299	0.0109	**0.1316** ***	-0.0009	5,010.637	4,886	0.2	0.2
**Cognitive proximity**	-0.0398	0.0275	**0.1006** ***	-0.0009	5,068.624	4,886	0.2	0.2

Sw size: Sliding window size; SW start: Starting point of the sliding window;

Note:

* p<0.1;

** p<0.05;

*** p<0.01

## 6 Discussion and limitations

### 6.1 Data

The low *F1-score* of both the *Twitter handle* and the *Website Detection Algorithm* is the result of low *Recall* values of these algorithms. However, this does not necessarily mitigate the statistical correctness and the meaning of the results. Since, in our analysis, high levels of *Precision* are more important to ensure that the compared connections, reflect actually valid connections.

Indeed, the *Precision* is 90.63% for the *Website Detection* and 100% for the *Twitter Handle Detection Algorithm*. Hence, both algorithms should provide sufficiently accurate results. This is also confirmed through the random sample of size 100 drawn from the 11,892 companies which were used for the final analysis. The *Precision* in this sample suggests that 97% of all connections in the Hyperlink and the Follower Network represent reliable connections. Hence, it could be concluded that the utilized 11,892 companies provide a representative sample of the 112,653 companies in the IT-sector. This conclusion is further substantiated by the fact that company characteristics appear to not influence the detection of a website or a Twitter handle at all.

Also, it should be noted that the overall accuracy of the *Website Detection Algorithm* is 71%, which is above conventional levels for company website discovery algorithms [53]. This could be a confirmation of the assumption that companies from the IT sector are more likely to have a website.

However, the comparison of the number of follower connections between companies which were detected and those which were not, revealed significant differences. **This in turn suggests that companies which are more active on Twitter, are detected more often by the utilized approach**. Which might be due to the fact, that such companies are more likely to directly link their Twitter account on their website to encourage engagement. **As a result the number of connections in the Follower Network is biased. Meaning, that the companies which were detected by the** ***Twitter Handle Detection Algorithm*** , **are potentially better connected on Twitter (entertain more Follower connections) than the average company would**. This has to be considered when interpreting structural differences and centrality measures such as degree and closeness centrality. Nevertheless, proximity measures might not be as susceptible to this distortion. Since, they are averaged over the amount of directly linked partners of a company and thereby not directly influenced by the amount of connections made by an individual company. Hence, comparing proximity measures still yields a reliable comparison between innovation associated connection patterns in the Follower and Hyperlink Network.

### 6.2 Methods

Two main choices regarding the methodology of this paper need to be addressed. **First**, the investigated company characteristics (sales, size and year) might not capture all possible influences on the connection behavior of innovative companies in the Hyperlink and Twitter network. However, these variables present a first starting point for the analysis. Specifically, since basic company characteristics (specifically size of a company) have been found to correlate with business innovation [48, 49]. This suggests that company characteristics closely related to its size such as sales and year of establishment, could also provide suitable variables. However, it remains the task of future research to provide an adequate set of company characteristics to appropriately examine company connection behavior associated with innovation.

**Second**, the definitions of the sliding windows utilized for the logistic regression models, need to be addressed. As mentioned earlier, the sliding windows and their starting points have been set in order to optimize the calculation of the logistic regression model. Meaning, they were adjusted to specifically account for differences of the particular investigated centrality or relational proximity measure. Nevertheless, the significance and the intensity of the coefficients, strongly depends on the size of the sliding windows in combination with the starting points. Therefore, the results of the logistic regression models should not be numerically interpreted. They are rather intended to illustrate that for a given definition of similarity, certain company characteristics can influence the chance of a company to behave according to similar connection strategies in both networks.

### 6.3 Results

#### 6.3.1 Structural differences

The results showed that the predominant structural difference between the Hyperlink and the Follower Network is the number of edge-connections. In particular, on average companies tend to form almost 21 times more follower connections. As mentioned above this difference has to be interpreted with care due to the bias of the *Twitter Handle Detection Algorithm* towards companies with more Twitter connections. Nevertheless, [35] similarly discovered that Korean politicians connected almost 4 times more on Twitter than via hyperlinks. However, in their study the Twitter network was considerably smaller than their hyperlink network. This was due to the fact that only 22 out of 99 Korean politicians had a Twitter account at that time. Therefore, it could be that 22 *a priori* more interactive politicians chose to have a Twitter account and thereby distorted the density in the Twitter network. **In contrast, the results in this paper are immune to such selection effects, since the same 11,892 companies are compared in both networks and the results confirm a considerably higher density for the Follower Network. Nevertheless, the bias of the** ***Twitter Handle Detection Algorithm*** **towards more active Twitter accounts remains, which suggests that the actual difference in edge-connections might be less pronounced**.

Furthermore, the max degree, the average in- and out-degree, the isolates and the clustering coefficients indicate that companies in the Follower Network are more broadly connected than in the Hyperlink Network. Meaning, they might not only follow specific key players, but also entertain more wide-spread links to different companies. Again, [35] observed similar connection patterns in their hyperlink and Twitter networks of Korean politicians. In particular, politicians engaged in more inter-party linkages on Twitter, which [35] ascribed to the facilitated access to information about an opponent’s political strategies. Meaning, politicians might follow adversaries in order to mimic or learn from their public policies [35]. Indeed, similar underlying motivations might be responsible for the connection patterns observed in the company Follower Network. This is due to the fact that social media networks are easily accessible and can serve as a marketing tool for companies. In particular, it allows them to interact with costumers and cultivate their corporate identity as suggested by [54]. **Hence, companies might follow their competitors more frequently on Twitter in order to study their consumer interaction or online marketing strategies**.

#### 6.3.2 Information flow patterns between companies

The out-degree and the out-closeness appear to be strongly dominated by the larger amount of edge-connections in the Follower Network. Specifically, the difference in out-closeness between the networks further substantiates the hypothesis introduced by [35]. **In particular, companies are more broadly connected on Twitter, which might further indicate that Twitter is also used to learn from competitors. Nevertheless, these observed effects might be exaggerated as a result of the biased** ***Twitter Handle Detection Algorithm*** **towards companies with more Twitter connections (see prior discussion of the data)**.

Beyond that, both networks have a relatively similar distribution of betweenness centrality. Accordingly, the effect size differences confirmed only a small difference between the means of the betweenness centrality distributions of the Hyperlink and Follower Network. **In turn, this suggests that connection patterns which capture the influence of a single company on information flow within the network, might be similar across the Hyperlink and Follower Network**. Again this is specifically interesting as such centrality measure are typically used to identify business innovation in offline company networks [5, 9]. Thus, the similarity of the innovation relevant betweenness centrality across both networks might suggest that innovative companies occupy similar positions in hyperlink and social media networks.

#### 6.3.3 Relational proximity measures

First of all, the density of the normalized geographic proximity in the Hyperlink Network bears no strong resemblance with the geographic proximity of German IT companies in the *Digital Layer* found by [9]. This result is not surprising and is probably attributable to the geographic differences between the United States and Germany.

Further, the scatter plot of the geographic proximity clearly reveals many companies with similar high geographic proximity in the Hyperlink and Follower Network. This is confirmed even more conclusively when limiting the focus to only those companies that maintain edge-connections in both networks (non-zero companies). **Specifically, the effect size differences between the geographic proximity means of non-zero companies across the networks turned out to be very small**. Also, it has been shown that the higher the geographic proximity of a company in one network, the more likely it has similar average edge distances in the other network. **Meaning, that connection strategies in the two networks are more likely to converge as the geographic distance bridged by a company increases**. This might indicate that connections which bridge larger geographic distances reflect a more serious intent of a company and are therefore mirrored across both network types. In this context, the results by [10] are specifically intriguing, as they observed that stronger long ranging hyperlink connections (i.e. connections embedded in cliques) in combination with weaker regional hyperlink connections (i.e. connections connecting different parts of a network) are in fact positively related with innovation. **This is a remarkable finding, as it suggests that hyperlink connection strategies associated with more innovative behavior can be similarly observed in the respective social media company network**.

In contrast to the geographic proximity, the density of the cognitive proximity in the Hyperlink Network bears more resemblance with the density of the cognitive proximity found by [9]. Furthermore, testing for effect size differences in cognitive proximity of non-zero companies revealed only a small mean difference between the Hyperlink and Follower Network. Hence, the effect size difference and the visible similarities reaffirm that non-zero companies behave according to similar connection strategies across both networks with regards to cognitive proximity. **Therefore, the knowledge base of a company appears to be of similar importance when linking via hyperlinks or follower connections**. This is in turn in line with the findings of several studies investigating innovation in offline [29, 55] or hyperlink company networks [9, 10], as they ascribe particular importance to cognitive proximity with regards to innovation. Hence, the similarities in connection behavior with regards to cognitive proximity might be a result of innovative companies aligning their connection strategies across both networks.

#### 6.3.4 Influence of company characteristics

The results of the linear regression models revealed a clear significant and positive effect of company size on various dependent variables. Note that the dependent variables examined in this paper capture at least some factors which are relevant for company innovation [9, 10, 29]. **Therefore, these findings are in accordance with [48, 49], as they found a clear positive relationship between a company’s size and its innovativeness**.

However, the coefficients found in the Follower Network in general tend to exceed those effects found for the Hyperlink Network. This can be attributed to the different network densities, similar to the results of [35]. Since, less edge connections in the Hyperlink Network lead to less variation in the dependent variable and thereby reduce the influence which explanatory variables can have.

Furthermore, the results of the logistic regression models suggest that the larger a company becomes or the higher its sales rise, the higher its chance to apply similar connection strategies in both networks. Considering, the positive correlation between company size and its innovativeness [48, 49], **it is reasonable to assume that companies which align their connection strategies across the Hyperlink and Follower Network could in fact be more innovative companies**. Hence, this might indicate that more innovative companies, among other things, align their online connection strategies. Nevertheless, the presented coefficients are not meant to be interpreted numerically. They are rather meant to showcase that similarities in connection behaviors between the two networks, can partly be explained by company characteristics.

## 7 Implications and future research

In general, this research contributes to a deeper understanding on how information propagates through different online company networks. Even though in total more connections are formed on Twitter, companies tend to align their connection strategies with regards to innovation relevant proximity measures such as geographical and cognitive proximity across both network types. Hence, the results so far indicate that social media company networks are able to reflect geospatial connection strategies of innovative companies similarly as their hyperlink counterparts. This opens new pathways for the analysis of company innovation with the help of social media and hyperlink networks.

In particular, the results encourage the development of a new theoretical foundation with regard to the definition, identification and prediction of business innovation using online social media networks. Similarly to the work of [10] for hyperlink networks, future research should further investigate specific connection patterns found in social media company networks and their relation to company innovation. This can potentially provide substantial insights into what motivates companies to form ties, collaborate and exchange knowledge, which in turn enables a more differentiated view on the driving factors of business innovation.

Note, given that future research substantiates the prior claims, a broad range of practical implications can possibly follow. In particular, assuming that social media company networks capture offline innovation processes comparably well as hyperlink networks, would offer a new and cost-effective alternative to conventional approaches when analyzing company innovation. This is due to the fact that social media network scraping is mostly enabled through APIs, which makes it in comparison faster and cheaper than hyperlink scraping. Also adapting connections on social media is not as expensive in terms of time and money for a company as on its website. Hence, social media networks could offer a comparably cheap and near real-time tool to assess company connections. In turn, this could allow researchers, policymakers and economic actors to faster identify additional incremental offline and online indicators for company innovation and further improve its prediction.

## 8 Conclusion

This paper sought to establish how (geospatial) connection strategies associated with company innovation, differ between hyperlink and social media company networks. For this purpose, the hyperlink and Twitter network of 11,892 U.S. IT companies were constructed and compared along four dimensions. First, basic structural differences between the networks were assessed. Second, traditional *SNA* methods were used to examine information flow patterns between companies. Third, in the spirit of [9], relational proximity measures were calculated. Fourth, the influence of company characteristics (size, sales and year) on connection patterns across different networks was investigated with the help of linear and logistic regression models.

Thereby, this paper revealed that on a general level structural differences between the networks seem to prevail and overall companies connect according to different motivations across the Hyperlink and Follower Network. In particular, almost 21 times more companies entertain edge-connections in the Follower Network than in the Hyperlink Network. Nevertheless, for those companies which do entertain edge-connections in both networks, similarities are apparent in terms of average geographic distance bridged (geographic proximity) and importance of the knowledge base of a partner (cognitive proximity). Additionally, company characteristics such as the size of company, exert similar influence on centrality and relational proximity measures across both networks. Further, larger company size and higher sales volume of a company also enhance its chance to connect according to similar strategies across the Hyperlink and Follower Network. Beyond that, the results suggest that it is most likely that those companies which align their connection strategies across both networks are in fact the most innovative ones. This leads us to the conclusion that the geospatial dimension and business innovation influences connection strategies across hyperlink and Twitter company networks in a similar fashion.

Therefore, this paper makes the first step towards unveiling in which ways the decision of a company to connect across social media networks might be influenced by business innovation and geographic distance. However, it remains the task of future research to further substantiate these findings with the help of innovation data on a company level.
